# Genetically engineered trees for plantation forests: key considerations for environmental risk assessment

**DOI:** 10.1111/pbi.12100

**Published:** 2013-08-05

**Authors:** Hely Häggman, Alan Raybould, Aluizio Borem, Thomas Fox, Levis Handley, Magnus Hertzberg, Meng-Zu Lu, Philip Macdonald, Taichi Oguchi, Giancarlo Pasquali, Les Pearson, Gary Peter, Hector Quemada, Armand Séguin, Kylie Tattersall, Eugênio Ulian, Christian Walter, Morven McLean

**Affiliations:** 1 Department of Biology, University of OuluOulu, Finland; 2 Syngenta Jealott's Hill International Research CentreBracknell, UK; 3 Departamento de Fitotecnia, Universidade Federal de ViçosaViçosa, Brazil; 4 Department of Forest Resources and Environmental Conservation, Virginia Polytechnic Institute and State UniversityBlacksburg, VA, USA; 5 Biotechnology Regulatory Services, United States Department of AgricultureRiverdale, MD, USA; 6 SweTree Technologies ABUmeå, Sweden; 7 State Key Laboratory of Tree Genetics and Breeding, Chinese Academy of ForestryBeijing, China; 8 Plant and Biotechnology Risk Assessment, Canadian Food Inspection AgencyOttawa, ON, Canada; 9 Gene Research Center, University of TsukubaTsukuba, Japan; 10 Centro de Biotecnologia, Universidade Federal do Rio Grande do SulPorto Alegre, Brazil; 11 ArborGenRidgeville, SC, USA; 12 School of Forest Resources and Conservation, University of FloridaGainesville, FL, USA; 13 Donald Danforth Plant Science CenterSt. Louis, MO, USA; 14 Natural Resources CanadaQuébec, QC, Canada; 15 Office of the Gene Technology RegulatorCanberra, ACT, Australia; 16 FuturaGene Brasil Tecnologia Ltda.São Paulo, Brazil; 17 ScionRotorua, New Zealand; 18 Center for Environmental Risk AssessmentWashington, DC, USA

**Keywords:** genetic engineering, forests, environment, risk assessment

## Abstract

Forests are vital to the world's ecological, social, cultural and economic well-being yet sustainable provision of goods and services from forests is increasingly challenged by pressures such as growing demand for wood and other forest products, land conversion and degradation, and climate change. Intensively managed, highly productive forestry incorporating the most advanced methods for tree breeding, including the application of genetic engineering (GE), has tremendous potential for producing more wood on less land. However, the deployment of GE trees in plantation forests is a controversial topic and concerns have been particularly expressed about potential harms to the environment. This paper, prepared by an international group of experts in silviculture, forest tree breeding, forest biotechnology and environmental risk assessment (ERA) that met in April 2012, examines how the ERA paradigm used for GE crop plants may be applied to GE trees for use in plantation forests. It emphasizes the importance of differentiating between ERA for confined field trials of GE trees, and ERA for unconfined or commercial-scale releases. In the case of the latter, particular attention is paid to characteristics of forest trees that distinguish them from shorter-lived plant species, the temporal and spatial scale of forests, and the biodiversity of the plantation forest as a receiving environment.

## Introduction

The United Nations Food and Agriculture Organization (FAO, 2010b) estimates the world's forest area at slightly >4 billion ha, which is 31% of the terrestrial land area. Forests are vital to the world's ecological, social, cultural and economic well-being. They play a major role in the global carbon cycle, storing 289 gigatons of carbon in biomass alone (FAO, 2010b), and protect soil and water resources, control avalanches, stabilize sand dunes, control desertification, protect coastal regions and provide habitats for many plants and animals. Wood is an important commodity. It is the raw material for lumber, pulp, paper, packaging, and increasingly a feedstock for bioenergy, biofuels and biomaterials. Forests provide the majority of fuel used for cooking and heating, and are important for recreation, tourism, and cultural and spiritual well-being.

The continuing increase in the world's population is raising demand for the goods and services from forests. Worldwide wood use increased from around 2.9 billion m^3^ in 1980 to around 3.4 billion m^3^ in 2010, with about half used for industrial purposes and half used as fuel for cooking and heating (FAO, 2010b). Wood use will likely continue to increase as populations expand, standards of living rise, and new uses for wood in bioenergy and biomaterials are developed. Large areas of forest are being converted to agriculture or urban development; approximately 13 million hectares of forest were lost between 2000 and 2010 (FAO, 2010b). Timber harvest is restricted in many forests, and those with high biological diversity where harvesting is prohibited now represent 13% of the world's forests (FAO, 2010b). Harvesting is also often prohibited in forests in sensitive watersheds, coastal areas or on the edge of expanding deserts to conserve soil and water. Only about 30% of the world's forests have a designated primary function of wood production, and the area of such forests continues to shrink, decreasing by more than 50 million hectares since 1990 (FAO, 2010b).

**Figure 1:**
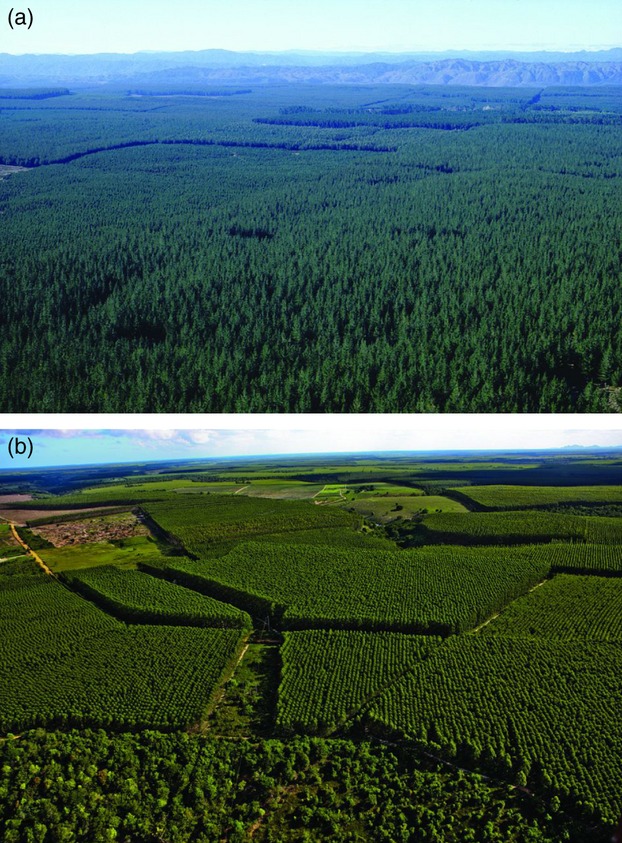
Plantation forests of (a) radiata pine in the Kaingaroa Forest, New Zealand; (b) eucalyptus in Bahia State, Brazil. Planted forests, composed of trees established through planting and/or deliberate seeding, comprise an estimated 264 million hectares or 6.6% of the total forest area and have the potential to produce almost two-thirds of current global wood production (FAO, 2010b). Photography credits: (a) Scion Photolibrary; (b) Ricardo Teles.

Intensively managed, highly productive forestry incorporating the most advanced methods for tree breeding is one solution to the problem of growing demand for wood and other forest products (Figure 1). Consequently, there is increasing interest in applying advanced molecular tools, including genetic engineering (GE), to improve the productivity or marketability of trees in commercial plantations (Harfouche *et al*., 2012). However, concerns have been expressed about the use of genetically engineered (GE) trees (e.g. Steinbrecher and Lorch, 2008), because of potential impacts to the environmental, economic, cultural and social services provided by forests.

Since the first regulatory approvals for cultivation nearly 20 years ago, GE plants have been grown commercially in 29 countries. Although these countries regulate GE plants using different procedures, the environmental risk assessment (ERA) paradigm is essentially the same for all. This paradigm, reviewed by (Hill, 2005; Nickson and McKee, 2002; OECD, 2003; OGTR, 2009; USEPA, 1992, 1998; Wolt *et al*., 2010), considers the biology of the crop (host) plant that has been genetically engineered, the characteristics of the introduced trait, the likely receiving environment (including relevant management practices in that environment) and their interactions.

This paper examines how the ERA paradigm used for GE crop plants may be applied to GE trees for use in plantation forests. It emphasizes the importance of differentiating between ERA for confined field trials of GE trees, and ERA for unconfined or commercial releases. In the case of the latter, particular attention is paid to characteristics of forest tree species that distinguish them from shorter-lived plant species, the temporal and spatial scale of forests, and the biodiversity of the plantation forest as a receiving environment.

### Understanding the receiving environment: what is a planted forest?

The worldwide average growth rate in natural forests is around 2 m^3^ per hectare per year (Clawson, 1975; South, 1999). At this level of productivity, approximately 1.7 billion ha of forest must be harvested annually to meet the world demand for wood. However, more wood could be produced by intensive management of forest plantations. In 2000, plantations comprised only 5% of forested land but contributed approximately 35% of the industrial wood harvested (FAO, 2001). Since then, the acreage devoted to planted forests has risen to 264 million hectares, representing 7% of the world's forests. From 2005 to 2010, the area of planted forests increased by about 5 million hectares annually, mostly through afforestation of previously nonforested land (FAO, 2010a). Approximately three quarters of planted forests consist of native species (FAO, 2010a). Improved plantation management, including site preparation, weed control, fertilization and utilization of improved genotypes, have substantially increased productivity and shortened rotations (growth and harvest cycles) in many regions (Evans and Turnball, 2004; Fox *et al*., 2007; Gonçalves *et al*., 2004). These practices are important considerations in the ERA of GE forest trees.

To sustainably realize the potential productivity in managed plantations, integrated silviculture regimes are needed where the tree crop, soil and other vegetation are actively managed to optimize growth (Fox, 2000). Implementation of these intensive regimes requires knowledge of how a tree's genetic make-up interacts with the environment to affect productivity, stem quality, wood quality and resistance to insects and diseases (Gonçalves *et al*., 2004). In addition, a site-specific understanding of what resources limit production (temporally and spatially) and how cultural treatments can be used to ameliorate these limitations are required (Allen *et al*., 2005). In many respects, intensive plantation silviculture is similar to agriculture, but is still firmly based on forestry's strong ecological foundations.

The potential gains in plantation growth and value through tree breeding are large (see Box 1). Due to the long breeding cycles of forest trees and time needed for progeny testing, many plantations are still planted with open-pollinated, half-sibling families (Hamilton *et al*., 2008; McKeand *et al*., 2003; Potts *et al*., 2008). However, even in these cases, the utilization of improved seed material can be economically viable exemplified by the case of Scots pine (Ahtikoski *et al*., 2012). Controlled pollination, where elite parents are bred together, is increasingly utilized because the resulting progeny show improvements in growth, stem and wood quality, and insect and disease resistance. Clonal forestry promises to increase the productivity of forest plantations further. Clonal *Eucalyptus* plantations, widely planted in the Southern Hemisphere, have dramatically improved productivity with growth rates exceeding 100 m^3^/ha/year in intensively managed plantations in Brazil (Evans and Turnball, 2004). Existing tree breeding, propagation and deployment practices (Box 1) may be indicative of how GE trees can be integrated into plantation forestry in the future.

**Box 1. Breeding, propagation and deployment practices in plantation forestry**

In the late 1940s and early 1950s, many countries began formal forest tree breeding and improvement programmes, starting with species identification trials that tested a variety of native and exotic species known to produce wood with mechanical and chemical properties suitable for making solid wood, pulp and paper products (White *et al*., 2007). Following species selection trials, wider ranges of natural genetic variation or provenance trials were established. The superior genetic individuals from these provenance trials were used to create the initial breeding populations. In the case of exotic species, this practice started the development of local landraces better adapted to local conditions. After these initial rounds of mass selection, most tree improvement programmes have followed recurrent selection, the most common strategy used for breeding of domesticated animal and crop species (White *et al*., 2007).

Breeding of forest trees has principally focused on improving economically and ecologically important traits including early stem growth and stem form, pathogen and disease resistance, adaptation and more recently wood properties. These traits show complex patterns of inheritance, and breeding programmes are also challenged by the long breeding cycles of tree species (White *et al*., 2007). In gymnosperm species, this is typically >15 years while in angiosperm species, the breeding cycles are typically shorter, but still take 7 years or more.

Forest species planted commercially contain broader genetic and phenotypic diversity than domesticated crop species. Large base and breeding populations (≥300) are used to maintain genetic diversity for long-term gain and to minimize the impact of inbreeding depression. To enhance genetic gain per cycle, vegetative propagation methods can be used to create multiple genetically identical copies or clones of individual progeny. Field tests with clones improve estimates of genetic values on which to base selection for future breeding populations (Baltunis *et al*., 2009; White *et al*., 2007). Hybrid vigour has been found and utilized in breeding of many angiosperm forest tree species particularly from the *Eucalyptus* and *Populus* genera (Potts and Dungey, 2004).

Operational deployment of improved germplasm is typically made with seed orchards and in select species by vegetative propagation methods (McKeand *et al*., 2003; Potts *et al*., 2008). Seedlings for operational planting are also produced by mass vegetative propagation methods, most commonly micropropagation, rooted cuttings and somatic embryogenesis (White *et al*., 2007). In angiosperm species, it is common for commercial forestry operations to plant rooted cuttings of well-characterized genotypes for clonal forestry as is the case for most commercial plantings of *Eucalyptus* in Brazil and Colombia and *Populus* in the US. For gymnosperms, significant amounts of rooted cuttings of *Pinus radiata* are produced for commercial planting in New Zealand and Chile. In addition to rooted cuttings, select gymnosperm species like *Picea glauca*, *Picea abies* and *Pinus taeda,* can be multiplied by somatic embryogenesis to produce clonal plants for commercial planting. For most forest tree species, commercial deployment is moving towards planting full-sibling families and better characterized, faster growing, vegetatively propagated clones. This trend is driven by technical advances for efficient, cost-effective scale up of full-sibling seed production and vegetative propagation, as well as the better returns for growers that faster growing, higher yielding families and clones produce (Potts *et al*., 2008).

## Environmental risk assessment of GE trees in confined field trials

The precommercial development of GE plants typically follows a series of steps, each accompanied by regulatory oversight (Figure S1). Confined field trials provide scientists with an essential experimental platform to further basic and applied scientific research through the evaluation of GE plants outside of the laboratory or greenhouse. Confined field trials also enable product developers to evaluate the performance of transgenic events and collect data to meet regulatory requirements.

The first ERA for a particular GE event is usually for a confined field trial. Regulatory permits to conduct confined field trials of a GE plant impose limits in time and space. These may include limits on the scale (e.g. area to be planted, number of locations and number of plants), the duration and the types of allowed activities (e.g. transportation, analytical and other experimental studies etc.). Permittees must apply control measures to ensure that the GE plants stay within these limits. As for GE crops, measures to confine GE trees may include spatial separation from the same or sexually compatible species, planting of border rows/pollen traps, and/or specific equipment cleaning, transport and disposal procedures.

ERAs for confined field trials primarily focus on the effectiveness of the risk control measures. Consequently, fewer data are needed to evaluate the risks posed by field trials than for commercial-scale environmental releases. Confined field trials of GE plants typically evaluate phenotypic characteristics and agronomic performance. The biology of the parent species, in particular, its reproductive capacity, and its potential for gene flow and long-distance dispersal, must be considered when determining the suitability and effectiveness of control measures. Details of the introduced gene(s)/genetic modification, the expected phenotype and any experience with the same or similar modifications in other species of plants may also be useful.

National information about the species and traits in confined field tests of GE trees is publicly available via the internet (Table 1). In some cases, a detailed description of the genetic modification and associated risk assessment are available. In the United States, risk assessments have been published for field trials of eucalypts, poplar, white spruce and sweetgum as well as nonforest trees including papaya, apple, walnut and plum (APHIS, 2012). Similarly, New Zealand has published detailed risk assessment and risk management decisions for field trials of GE trees (NZEPA, 2012). In Australia, while no GE forest trees have been approved for field trials, detailed risk assessment and risk management plans are available for several fruit trees and other long-lived perennials, including papaya, banana, grapevine and sugarcane, that are relevant for forest trees (OGTR, 2012b). In the European Union, Notification Reports are published that summarize potential environmental impacts and risk management measures (JRC, 2012). Notably, these reports specifically address forest trees as recipient or parental plants and describe factors affecting potential dissemination. Countries including Japan (J-BCH, 2012) and Brazil (CTNBio, 2013) provide information on the activities and procedures implemented to manage risk, while Canada publishes species-specific terms and conditions that must be met while conducting confined field trials (CFIA, 2012).

**Table 1:** Summary of confined field trials (CFTs) approved for genetic engineering forest trees in different countries indicating where risk assessments are available to the public

Country or region	No. of CFTs approved	Forest tree species approved for CFTs (# per species)	Risk assessments publicly available	Source
United States	500	*Populus* spp. (212), *Pinus* spp. (154), *Eucalyptus* spp. (77), *Liquidambar styraciflua* (37), *Castanea dentata* (15), *Ulmus americana* (5), *Picea glauca* (1)	Yes	http://www.aphis.usda.gov/brs/status/BRS_public_data_file.xlsx
China	78	*Populus* spp. (34), *Robinia pseudoacacia* (25), *Larix* spp. (16), *Paulownia* (3)	No	M.-Z. Lu, Chinese Academy of Forestry, pers. commun.
Brazil	65	*Eucalyptus* (65)	No	http://www.ctnbio.gov.br/index.php/content/view/3509.html
Canada	45	*Populus* (28), *Picea mariana* (10), *P. glauca* (7)	No*	http://www.inspection.gc.ca/english/plaveg/bio/confine.shtml#sum
EU	44	*Populus* spp. (30), *Betula pendula* (6), *Eucalyptus* spp. (4), *Picea abies* (2), *Pinus sylvestris* (2)	Yes	http://gmoinfo.jrc.ec.europa.eu/gmp_browse.aspx, http://gmoinfo.jrc.ec.europa.eu/overview/
Japan	9	*Eucalyptus* (7), *Populus* (2)	No	http://www.bch.biodic.go.jp/english/e_index.html
New Zealand	5	*Pinus radiata* (5)	Yes	http://www.epa.govt.nz/new-organisms/popular-no-topics/Pages/GM-field-tests-in-NZ.aspx
Australia†	0	N/A	Yes	http://www.ogtr.gov.au/internet/ogtr/publishing.nsf/Content/ir-1

* Canada publishes species-specific terms and conditions for managing field trials.

† While no forest tree species have been approved for field trials in Australia comprehensive risk assessments are available for a variety of other tree or perennial species.

### Key considerations for ERA of GE trees in confined field trials

For ERA of confined field trials of GE trees, a crucial aspect of the ERA is determining the effectiveness of confinement. The impact of the confined release of a GE tree on the environment will be minimized by the generally small scale of the release. Restricting access by wildlife or humans, and postharvest management and monitoring requirements, also contribute to minimizing potential environmental impacts. To determine the effectiveness of risk mitigation measures, a number of key aspects need to be considered: the reproductive biology of the host species; the biology of any sexually compatible species also present in or proximal to the receiving environment; potential for long-distance dispersal of propagules; and ecological interactions particularly if the interactions are with species of concern such as protected species or weeds. Information about the receiving environment including silvicultural practices may also be useful.

Reference biology documents, such as those published by the OECD and a number of individual countries, are available for many plant species including several trees (Craig *et al*., 2008; OECD, 2006). These simplify access to essential information about the reproductive biology of the host organism. Additionally, the possibility of dispersal of propagules by humans and animals is addressed. Tree species that are currently used for plantation forest production have been well studied, and extensive information exists that can contribute to proposing and assessing effective strategies for confining field trials of GE trees (Brunner *et al*., 2007; Byrne, 2008; Henry, 2011; OECD, 2012).

It is then important to consider how the introduced trait, both in its intended and potential unintended effects, might alter the biology of the tree with respect to the ability to achieve adequate confinement. Several sources of information can be used to inform the risk assessment. For example, observations of the tree's phenotype from laboratory or greenhouse studies will be available for use in the risk assessment, and experience with the same or similar genes introduced into crop plants can enhance a risk assessor's prediction of the potential effects (e.g. Nickson, 2008; Wolt *et al*., 2010). Additionally, the objectives for development of many GE trees involve the same traits as those sought through traditional breeding. The potential environmental impacts of these traits will be the same, or similar, to those introduced by conventional improvement programmes, and so risk assessments should be informed by experience with conventionally derived traits (NAS, 1987; NRC, 1989; OECD, 1993).

The proposed receiving environment for a confined GE tree release needs to be considered case by case, just as required for other GE plants. Consequently, the ERA should consider characteristics specific to the proposed site such as environmental conditions, silvicultural practices and the presence of sexually compatible species in so far as they relate to the confinement of the trial. The focus of the ERA for field trials should be assessment of the likelihood that the conditions of the trial will confine the genetically engineered trait. It should not be necessary, and indeed it may be impossible, to make precise predictions about what might happen should the transgenes escape confinement. The purpose of the field trial is to collect data to help make such predictions while minimizing the probability of escape. Lack of detail about ecological interactions between the GE tree and the environment ought not to prevent a field trial if confinement is reliable. Proximity of the proposed trial site to protected species is an issue that should be addressed and may be a legal requirement (e.g. Australia's *Gene Technology Act 2000*; US' 7 CFR 340 and 7 U.S.C. § 136, 16 U.S.C. § 1531 et seq., Canada's Part V, Seeds Regulations, Brazil's Normative Resolution No. 5).

The scale and duration of a field trial affects its confinement and should be driven by the need for data to test particular hypotheses. While most crop plants are annual or can be made to complete an annual reproductive cycle, forest tree species typically take years to attain sexual maturity. This prolonged juvenile phase may be advantageous for confinement; performance and some biosafety-related data may be collected without measures to prevent dissemination of propagules. However, repeated reproductive cycles at maturity and tree longevity suggest that dispersal of propagules must be managed in later stages of the trial. In this respect, trees and herbaceous perennial plants such as alfalfa present similar risk scenarios. The size and woody nature of trees may result in the need for measures for disposal of GE plant residue additional to the common practices of incorporation into the soil, burial or incineration that are applied to herbaceous GE plants.

If there is insufficient information about the parent tree species or about the phenotype and anticipated behaviour of an experimental GE tree in the environment, measures to reproductively isolate confined field trials of GE trees might be needed. These measures mimic those applied to crop plants and include removal of the developing inflorescences from any early flowering individual trees, or termination of the trial prior to flowering. Monitoring of the trial site after trial completion is also a standard management measure to ensure that the GE trees do not re-establish. For example, root suckering is typical for several tree species including aspen species (Fladung *et al*., 2003), and vegetative regeneration with sprouting and coppicing is a natural characteristic of birch (Koski and Rousi, 2005).

It is a common misunderstanding that confined field trials and commercial releases are subject to essentially the same risk assessment. The confinement of these trials (described above) blocks many of the potential pathways to harm. Therefore, an ERA for a field trial permit can be completed without the exhaustive data that are typically needed for an environmental release without confinement. This is important because the field data that may be required to assess the risks of an unconfined release are usually generated from confined field trials. Confusion between data requirements for confined and unconfined releases has been exacerbated by the Cartagena Protocol on Biosafety (SCBD, 2000) that does not differentiate these activities and, consequently, many national biosafety regulatory frameworks developed to implement the Biosafety Protocol do not either. GE tree development cannot advance past the laboratory stage unless biosafety regulatory systems are able to permit the field evaluation of GE plants of uncertain risk (McLean *et al*., 2012).

## Environmental risk assessment of GE trees for commercial release

As previously mentioned, the paradigm for ERA of GE plants considers the biology of the host plant, the characteristics of the introduced trait(s), the intended receiving environment and interactions between these to estimate the likelihood that a field trial or cultivation will cause ecological harm. When applied to the ERA of GE plants for an unconfined release, the context of the assessment is very different than for confined field trials as limits to cultivation in time and space are generally not required (other than those practices that are normally applied when growing the conventional counterpart). Consequently, an ERA for an unconfined release of a GE forest tree will usually require significantly more information than for the ERA of a confined field trial in order to assess potential risks that will no longer be managed through confinement measures including: potential consequences of introgression of the transgenic trait into sexually compatible populations; and, impacts of longer-term exposure on organisms in the receiving environment. Data accrued from laboratory studies and confined field trials are supplemented with information from the literature and past risk assessments (where relevant) to address the likelihood that the new phenotype will cause harm. Commercial applications in fruit trees or other woody perennial species may also provide useful information relevant to the risk assessment of plantation forest tree species. Published risk assessments for papaya events (APHIS, 1996, 2009) and one plum event (APHIS, 2007a) are available, as are risk assessments for rose, another woody perennial species approved for commercial production in the United States (APHIS, 2011) and Australia (OGTR, 2012a). While the biology of these species differ in several aspects from that of forest tree species, these analyses serve as examples of how risk assessments have been successfully conducted for plants other than annual row crops.

### Biology of the host plant

Much is known about the biology of tree species that are being considered for GE and deployment in planted forests. Generic biology descriptions, like those published by the OECD, can be supplemented with country-specific information, such as the occurrence of sexually compatible species, which may not be included in source biology documents.

It is vital that existing data are fully exploited for ERAs of GE trees to avoid unnecessary delay and expense. As for GE crops, data on the biology of non-GE plants of the same species should be used to predict the likelihood of harm arising from cultivation of a GE tree. First, information on the types of harm caused by non-GE trees, such as adverse impacts on water tables or fire regimes, can help to define the types of harm likely to result from GE trees (NRC, 2002). Secondly, the ecology of a GE tree is likely to be predictable from the ecology of a non-GE tree. For example, knowledge of how genetic variation in traits such as lignin quality or quantity affects the ecology of non-GE trees may help to predict the ecology of trees with GE changes to lignin quality (e.g. Stackpole *et al*., 2011). Such studies may be particularly valuable to risk assessors if they show the genetic variation for the trait of interest and what, if any, effect on tree ecology can be observed (e.g. O'Reilly-Wapstra *et al*., 2005). Simulation of GE traits may also give useful information. For instance, the effects of insect or pathogen resistance can be simulated by excluding pests and pathogens from experimental plots. Such methods have been informative for predicting the effects of invasive species (Liu and Stiling, 2006).

### Characteristics of the introduced traits

Commercial release of GE trees has been extremely limited to date (see Box 2), but a review of the GE events in confined trials (see Table 1) provides insight as to the traits that regulatory authorities must consider in the near to medium term. Some of these traits are unique to GE trees (e.g. modified wood properties), whereas others are variations on the input traits that are common in herbaceous crop plants. The latter include herbicide tolerance, and traits to mitigate the impact of abiotic and biotic stressors. Existing knowledge about the environmental safety of proteins can and should be applied to the ERA of GE trees expressing the same or similar traits.

**Box 2. Commercial cultivation of GE poplar**

**Figure 2:**
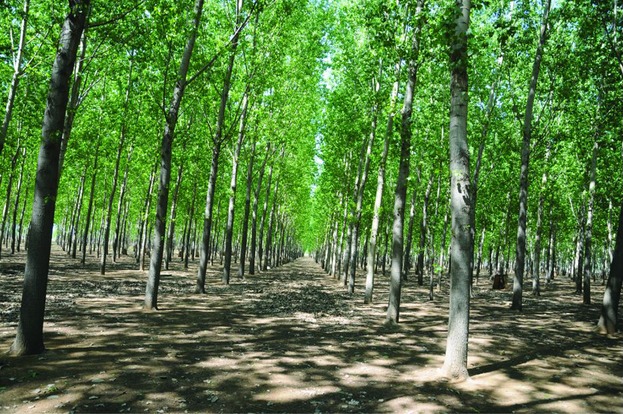
Transgenic poplar plantation in Huairou, Beijing, China.

To date, the only known approvals for commercial-scale cultivation of a forest tree species have been granted in China where two varieties of insect-resistant poplars have been planted since 2002 with one variety planted at eight sites in seven provinces (FAO, 2010a). The first variety is *Populus nigra* transformed with the *cry1Ac* gene from *Bacillus thuringiensis* (Bt). Three clones were selected for field testing, and one of these was commercialized and planted in northern China (Figure 2). The second variety is a hybrid white poplar, clone 741, transformed with a fusion of *cry1Ac* and *API* (coding for a proteinase inhibitor from *Sagittaria sagittifolia*). By 2011, these varieties occupied a total of 490 ha. The transgenic *P. nigra* has also been used for hybridizing with nontransgenic *Populus deltoides* to generate an insect-resistant source in a breeding programme designed to generate new hybrid clones, which could expand the planting area of Bt poplar clones.

#### Growth and wood properties

Wood (secondary xylem) functions in long-distance transport of water and nutrients from the roots to the shoots and provides mechanical support of the crown (Tyree and Zimmermann, 2002). The structure and function of wood in living trees is controlled by anatomical, chemical and mechanical properties, which also are vital to the utilization of wood for solid wood, pulp, paper and energy (Peter, 2007). The inter-relationships between growth and wood properties are central, as trees grown on commercial plantations need to produce high stem biomass and have wood properties suitable for conversion into renewable products (Mansfield *et al*., 2012; Peter, 2007; Vanholme *et al*., 2010; White *et al*., 2007).

Wood properties of forest tree species have wide natural genetic variation and are typically under stronger genetic control than growth traits (White *et al*., 2007). Wood is composed of about 25% lignin, and 70% cellulose-based carbohydrates, including approximately 45% cellulose and 25% hemicellulose (Sjöström, 1993). The most intensively studied property is wood lignin content and subunit composition, because of the potential economic impact on pulp, paper and bioenergy production (Baucher *et al*., 2003; Peter, 2007; Peter *et al*., 2007; Pilate *et al*., 2002; Simmons *et al*., 2010). In different tree species, the lignin content of wood has been shown to vary from 15% to 40% (Sarkanen and Ludwig, 1971). For example, in wild *Populus trichocarpa*, collected from across its natural geographic range, wood lignin content varies from 15.7% to 27.9% (Studer *et al*., 2011). Sykes *et al*. (2008) found more variability in lignin content in *Populus* species from ring to ring than at different heights going up the stem, indicating the importance of positional considerations when sampling trees to characterize biomass. In interspecific hybrids of *Eucalyptus* and *Populus*, tree growth and wood lignin content are negatively correlated (Novaes *et al*., 2010), suggesting that reducing lignin content will promote growth in these species. Multiple genes coding for enzymes in the monolignol biosynthetic pathway have been extensively studied and the impacts of natural and induced genetic variants (MacKay *et al*., 1997; Pedersen *et al*., 2005; Studer *et al*., 2011) and targeted down-regulation by GE characterized in numerous annual herbaceous, perennial grass and woody species (Boerjan *et al*., 2003; Fu *et al*., 2011; Kitin *et al*., 2010; Leplé *et al*., 2007; Studer *et al*., 2011; Vanholme *et al*., 2008; Voelker *et al*., 2010, 2011a,b) have been studied in greenhouse and field experiments. Consequently, extensive information exists on the effect of lignin modification on growth, pathogen and stress tolerance in a wide variety of model and crop plants (Pedersen *et al*., 2005).

A central consideration in each ERA is the potential effect of introgression of the modified trait on the natural population's fitness and function. Quantitative and population genetic theory postulates that traits most positively correlated with fitness of the species will show little to no genetic variation, as strong selective forces lead to fixation of alleles at these loci. Wood properties of forest tree species have wide natural genetic and phenotypic variation. Altering wood chemistry through GE by up- or down-regulating endogenous genes in the lignin, hemicellulose or cellulose biosynthetic pathways is likely to produce GE trees with wood properties found within this broad range seen in natural populations (see Table 2; Steane *et al*., 2011; Studer *et al*., 2011). If wood properties fall outside the natural range of variation, risk assessors may consider how natural genetic variation in these traits affects the ecology of non-GE trees as a predictor of the potential ecological impact of the GE tree. However, additional studies designed to evaluate the fitness of such GE trees may also be warranted on a case-specific basis.

**Table 2:** Potential impacts of changes in wood biomass and quality on forest tree growth, fitness and function

Trait	Potential impact: gymnosperm	Potential impact: angiosperm
Reduced lignin	If too low could limit growth and decrease fitness; may affect insect and disease* resistance.	If too low could limit growth and decrease fitness; may affect insect and disease resistance†
Increased syringyl lignin	Novel trait in gymnosperm wood, making the wood chemistry like angiosperms and improve utilization and processing of coniferous biomaterials‡	Improved pulping and biofuel production, may reduce growth and decrease natural degradation by fungi§
Increased stiffness	No change or slight increase in susceptibility to high wind damage¶	No change or slight increase in susceptibility to high wind damage**
Increased carbohydrates	Increased wood degradation during natural decomposition	Increased wood degradation during natural decomposition
Increased wood density	May affect growth††	No change‡‡
Increased lignin	Decreased growth, slower wood degradation during decomposition§§	Decreased growth, slower wood degradation during decomposition§§

* Wagner *et al*. (2009, 2012).

† Novaes *et al*. (2010).

‡ Wagner *et al*. (2012).

§ Giles *et al*. (2012), Hancock *et al*. (2007), Stewart *et al*. (2009) and Wagner *et al*. (2009).

¶ Barnett and Bonham (2004).

** Kitin *et al*. (2010), Voelker *et al*. (2011a,b).

†† Khan (2012) and Peltola *et al*. (2009).

‡‡ Zanne *et al*. (2010).

§§ Novaes *et al*. (2010).

#### Insect and disease resistance

Insect-resistant transgenic crops transformed with endotoxin genes derived from the soil bacterium Bt are amongst the most common of the commercially available transgenic plants used in agriculture today. Similar strategies have also been applied to forest tree species. More than two decades ago, hybrid poplar with resistance towards two lepidopterous pests, forest tent caterpillar and gypsy moth, was developed (McCown *et al*., 1991). Other poplar hybrids have also been engineered with Bt endotoxins, and an overview of the phenotypes observed is provided in Confalonieri *et al*. (2003). Stability of the insect resistance trait in transgenic *P. nigra* plantations in China (see Box 1) has been reported based on observations from field studies conducted in the Manasi Plain Forest Station, Xinjiang Uygur Autonomous Region, from 1994 to 1997 (Hu *et al*., 2001) and 1997 to 2001 (Hu *et al*., 2007) where leaf defoliation caused by *Apocheimia cinerarius* and *Orthosia incerta* was 10% in *P. nigra* plantation and up to 90% in nontransgenic poplar plantations. Insect resistance using a Bt endotoxin gene was also achieved in *Eucalyptus* (Harcourt *et al*., 2000). As an alternative to Bt, some laboratories have investigated the efficacy of various proteinase inhibitors to confer insect resistance in poplars (reviewed in Confalonieri *et al*., 2003), and the manipulation of tryptophan decarboxylase (Gill *et al*., 2003). Several conifers are also susceptible to insect forest pests, and resistant GE loblolly pine (Tang and Tian, 2003), radiata pine (Grace *et al*., 2005) and white spruce (Ellis *et al*., 1993; Lachance *et al*., 2007) have been developed.

Relatively few publications have reported enhanced disease resistance in GE forest tree species. Poplars have been engineered to improve resistance to the fungal pathogen *Septoria musiva* by increasing the production of oxalate oxidase (Liang *et al*., 2001), or to resist *Melampsora medusae* by expressing an exogenous endochitinase (Noël *et al*., 2005). A field trial with transgenic birch demonstrated that expression of a chitinase gene limited damage caused by the birch rust (fungal) pathogen (Pasonen *et al*., 2004). Black spruce engineered with a *Trichoderma* endochitinase showed reduced disease symptoms to the root rot disease causal agent (Noël *et al*., 2005). American elm has been engineered to enhance Dutch elm disease resistance by targeted vascular expression of a recombinant antimicrobial peptide (Newhouse *et al*., 2007), and chestnut blight resistance has been improved by introduction of an oxalate oxidase gene in American chestnut (Welch *et al*., 2007). A number of confined field trials are underway in the US specifically looking at Dutch elm disease resistance in American elm and chestnut blight resistance in American chestnut (Thompson, 2012).

A limited number of studies have assessed if increased resistance to microbial pests impacts beneficial symbiotic microbes such as mycorrhizal fungi. The results with the expressed traits studied, showed limited or no effect on soil fungal communities, or the forming of symbiotic interaction with GE trees (Lamarche *et al*., 2011 and references therein; Stefani and Hamelin, 2010).

#### Abiotic stress tolerance

Although natural abiotic stress tolerance involves the complex interaction of many genes, research has shown that overexpression of single genes can render plants tolerant to specific abiotic stressors (Bhatnagar-Mathur *et al*., 2008; Khan, 2012; Mittler and Blumwald, 2010; Reguera *et al*., 2012). These genes encode a variety of proteins including (i) osmoprotectants or osmolytes like polyamines, (ii) aquaporins (membrane water transport proteins) and membrane ion transporters, (iii) enzymes like ascorbate peroxidases, which act as scavengers of reactive oxygen species, (iv) chaperones, heat-shock proteins, dehydrins and other proteins that help to repair damaged proteins and/or prevent protein aggregation during desiccation, (v) transcription factors involved in the regulation of stress-responsive genes, (vi) proteins of the hormonal response machinery, especially those related to abscisic acid and cytokinins, the two phytohormone classes most crucially regulating abiotic stress response (Bhatnagar-Mathur *et al*., 2008; Mittler and Blumwald, 2010; Reguera *et al*., 2012).

Examples of GE trees expressing abiotic stress-tolerance genes have been recently reviewed (Gambino and Gribaudo, 2012; Harfouche *et al*., 2011; Osakabe *et al*., 2012; Yadav *et al*., 2010). Some of the most promising abiotic stress-tolerant GE trees intended for timber production include pines, poplars and eucalypts (see Table 3).

**Table 3:** Examples of abiotic stress-tolerance research in genetic engineering forest tree species

Species	Trait	References
*Eucalyptus*	Freeze tolerance	Mizoi *et al*. (2012) and Zhou *et al*. (2011)
*Populus tremula* × *Populus alba*	Freeze tolerance	Benedict *et al*. (2006)
*Pinus virginiana*, *Pinus strobus*	Tolerance to drought, freezing, and salt	Tang *et al*. (2007)
*Populus alba* × *Populus berolinensis*	Salt tolerance	Li *et al*. (2009)
*Eucalyptus globulus*, *Eucalyptus camaldulensis*	Salt tolerance	Kikuchi *et al*. (2006, 2009), Yu *et al*. (2009, 2013a,b)

### Receiving environment

In addition to the host plant and trait(s), the receiving environment where GE trees are expected to be deployed must also be taken into account as part of the assessment. This includes consideration of areas where the GE trees may be grown or reach through dispersal; potential scale of the release, which is dependent upon degree of adoption; environmental conditions; habitat, climate and soil suitability; land use in the area; standard silvicultural practices and how they might differ for the GE tree species (e.g. changes in use patterns of pesticides in plantations of GE trees modified for resistance to insect pests); presence of sexually compatible plants; presence of vulnerable or susceptible entities; and presence of similar genes in the population.

### Key considerations for ERA of GE trees for unconfined release

#### The selection of appropriate comparators

ERAs of GE plants for the purposes of unconfined or commercial releases are conducted using a comparative approach. The potential adverse environmental impacts of the GE plant in the receiving environment are compared with those posed by the unmodified host plant (OECD, 1993; OGTR, 2009; SCBD, 2000). This approach is essential because it permits the risk assessor to focus on the potential adverse effects of any identified differences between the GE plant and its unmodified (or conventional) comparator instead of trying to describe every potential environmental interaction that might occur between the GE plant and the environment (OECD, 2003). If the GE plant exhibits no differences in environmental interactions from the unmodified host plant other than the change conferred by the new trait, the ERA should focus only on the potential environmental impact of that change. If the change conferred by the new trait falls within the normal range and variation for that trait in the host species, then the risk assessment need not proceed further.

The choice of comparator can have a significant impact on the data requirements, interpretation and conclusions drawn from the risk assessment. Comparators chosen should be those that will provide the most relevant data. Suitable comparators should also encompass the normal range and variation for the species to differentiate inherent characteristics from those that derive from the introduction of the new trait. In practice, the counterpart used is usually the near-isogenic parent. In some cases, the most closely related comparator may itself be a transgenic plant, such as a subsequent transformation of an already commercialized transgenic plant. Generally, for comparative assessments, the genetic variability of the species will also be considered as part of the ERA, usually by considering a range of varieties, cultivars or lines in the case of crop species. Relevant data about genetic variability may already exist in the literature, and hence, it is important to note that comparative assessment does not necessarily imply comparative testing. Consideration of genetic variability will be particularly relevant for forestry species where natural variability is generally higher, as extensive breeding for variety development has not taken place. A sound knowledge of the biology of the comparator is central to the risk assessment because it is important to distinguish the potential for harm that arises as a consequence of the introduced traits from characteristics that are inherent to the species.

More data may be required when the GE tree represents a species new to production or new to the region of proposed deployment. In some cases, knowledge gaps will need to be filled with fundamental research, but there may still be the possibility of building on existing knowledge of the nontransformed host plant. Experience with the species in its native range can often be extrapolated to the receiving environment under consideration. When the biology of species related to the host plant is known, it can be a useful predictor of potential environmental interactions. Precaution can be applied to select worst-case scenarios when there are significant unknowns with regard to basic biology or environmental interactions. Conservative approaches are most relevant when the tree has shown invasive or weedy characteristics in its home range and there are a number of approaches that can be used to determine whether the unmodified proposed comparator is likely to be a weed (e.g. FAO, 2011; Virtue and Melland, 2003; Virtue *et al*., 2010).

#### Longevity

In considering the ERA of GE trees, it is frequently noted that trees are long-lived, and their exposure to the environment occurs over a much longer time than annual crops (Ahuja, 2009, 2011). However, trees are not unique in this regard, and work with perennial crops as well as continuous year over year planting of the same annual species can provide useful insights into long-term environmental exposures of both GE and conventionally improved trees. Indeed, risk assessment of annual crops assumes a prolonged (although in many cases seasonal) use of the crop. The persistence of trees over many years is also a characteristic of perennial crops such as grapes, coffee or tea. Vegetative propagation of these crops also parallels that of trees, as do more herbaceous species such as cassava, sweet potato, sugarcane and potato.

The presence of the tree in the environment for an extended period is not a risk, in and of itself. The impacts of longevity of a GE tree should be considered case by case, in conjunction with the tree's intended use including associated silvicultural practices, and specific receiving environment. The potential for adverse environmental effects from long-term exposure should be considered in the context of plausible and well-developed risk hypotheses, and the assessor should be able to identify what types of data would be useful in characterizing these.

#### Scale

Spatial scale is an important consideration when assessing the potential impacts of GE crops; this is also true for extensive plantings of GE trees on the environment. This may be best addressed through modelling, given the short time frames that field studies are usually conducted compared to the typical life cycle of the tree (IFB, 2008). Conducting large-scale, confined field studies to full rotation or maturation for many forest tree species is impractical, and modelling to deal with scale can be an integral part of the risk assessment process.

Extensive plantations of GE (and non-GE trees) may impact hydrology and/or soil nutrition. Large-scale deployment of forest trees such as *Eucalyptus* (Albaugh *et al*., 2013) as well as certain other crops, like *Miscanthus* (Vanloocke *et al*., 2010) and short rotation woody species, can have impacts on hydrology and soil nutrition (McIsaac *et al*., 2010). When it is anticipated that a GE tree will lead to increased growth rates or acreages in comparison with its conventional counterparts, then potential impacts on hydrology and soil nutrition can be addressed in the ERA. In this case, modelling based on field-level data can be used to extrapolate potential impacts over larger areas (Vose, 2012). The results of these models can also be used to determine which, if any, mitigation methods might be available to minimize any adverse impacts.

Other scale-dependent considerations can include (i) the potential consequences of introgression of the transgene in sexually compatible relatives, or (ii) large-scale plantings of clonally propagated trees that are genetically homogeneous and potentially more vulnerable to pests and diseases than a heterogeneous population. In the case of the former, the assessment will consider how a transgene may affect the population dynamics of these relatives (e.g. whether there are large increases in population growth rates or dispersal to new sites) and weed risk assessment models, which are in practical use in Australia, New Zealand and the USA (Cock, 2003), may be useful to predict likely invasiveness of GE trees (P. Keese, pers. commun.). In the case of the latter, the same types of data that are collected in non-GE tree trials that also deploy clones can be used to inform the risk assessment.

#### Assessing unintended effects

Many studies of GE crops have tested for unintended changes that may result from GE. Reviews of such studies have shown that GE is no more likely to introduce potentially harmful unintended changes than are other methods of introducing genetic variation into crops, such as hybridization and mutagenesis (Ricroch *et al*., 2011). As a consequence, it may no longer be necessary to conduct compositional analyses of key endogenous components of GE crops for risk assessment, except in cases where changes in composition are the intended effect of the trait, or where there is a plausible hypothesis about how the trait may change composition (Herman *et al*., 2009). The same conclusions should apply to GE trees, particularly as there is unlikely to be a need to measure GE trees for potentially harmful unintended nutritional changes for human health risk assessments. Thus, rather than a typical compositional analysis that measures key nutrients, measurement of unintended effects should focus on compounds that are known to be produced by the nontransgenic counterpart and that also meet the following three criteria: the compound has an ecological effect, there is a plausible mechanism by which the ecological effect could lead to environmental harm and there is a plausible mechanism by which the GE could lead to environmentally harmful changes in the concentration of the compound more frequently than by other methods of tree breeding or than by forest management.

#### Use of existing data

If a GE tree that produces a transgenic protein identical or sufficiently similar to one produced in a commercial GE crop, the ecological effects data used for the ERA of the GE crop may be informative for the ERA of the GE tree. If the protein is pesticidal, its effects are assessed on surrogate species that represent valued functional and taxonomic groups of organisms likely to be exposed to the protein via cultivation of the GE crop. These organisms are exposed to purified protein under standard conditions in the laboratory (Romeis *et al*., 2011). The ecological risks are estimated by comparing the effect of the protein in the studies with predicted worst-case exposures to the protein following cultivation of the GE crop. Negligible risk may be concluded if the highest concentration at which no adverse effect is observed (the no observed adverse effect concentration or NOAEC) is greater than or equal to the worst-case exposure via cultivation (e.g. Raybould and Vlachos, 2011). Provided the ecological effects data sufficiently cover the range of valued taxonomic and functional groups exposed to the protein via the GE tree, and also provided exposure to the protein via the GE tree is not significantly greater than via the crop, negligible ecological risk may be concluded for the GE tree without collecting further ecological effects data (Romeis *et al*., 2009).

For nonpesticidal traits, negligible ecological risk of GE crops is usually concluded from arguments about the mode of action of the protein (e.g. Raybould *et al*., 2010). Data and conclusions from ecotoxicological studies of insecticidal proteins expressed in annual crops could be applied to the same protein when expressed in a GE tree. Indeed, laboratory studies may be more important sources of data for GE trees, as the necessary replication required to generate ecological effects data from confined field studies would make such studies extremely difficult.

Data, strategies and models of ERA for abiotic stress-tolerant GE plants, particularly crops, have been reviewed, and these are equally informative for the ERA of abiotic stress-tolerant GE forest trees (Grumet *et al*., 2011; Khan, 2012; Nickson, 2008). As discussed in Khan (2012), abiotic stress tolerance may be considered a fitness enhancing trait when it increases reproductive and vegetative growth, and the competitive ability of plants subjected to selection pressure. In theory, increased fitness under stress conditions may in turn confer persistence or volunteer potential in agricultural lands and invasiveness in natural habitats. However, experience with crops improved for abiotic stress tolerance by conventional breeding shows that none have been found to have increased persistence, invasiveness or weediness. Because GE of abiotic stress-tolerance traits is being primarily applied to the same stress-related physiological and metabolic systems as with conventional breeding programmes, the ability of GE plants to become weeds, attain increased persistence or volunteer potential in agriculture, and invasiveness in natural environments as a result of improved abiotic stress tolerance is likely to be within the same range as for traditional breeding. Studies of a number of abiotic stress-tolerant crops and trees including maize, sugarcane, barley, cotton, soybean, wheat and *Eucalyptus* have led to the conclusion that the use of abiotic stress-tolerance genes in GE plants does not necessitate additional considerations for ERA when compared to herbicide or insect-resistant plants (reviewed in Khan, 2012).

#### Uncertainty in ERA

Although risk assessment is a scientific endeavour, it is important to remember that it is not basic research. Risk assessment is a structured, rational approach to address uncertainty based on the plausibility and strength of scientific/technical evidence. It is important that an assessment acknowledge the sources of uncertainty that are likely to have a significant impact on the likelihood and magnitude of identified risks. However, it is equally important that the assessment present these uncertainties in the context of the information and experience that is available for informing the assessment. It is also essential to distinguish scientific uncertainty from policy uncertainty in ERA as the latter can be confused for the former when decision-making criteria are neither defined nor transparent (Raybould, 2012). Highlighting uncertainty without putting it into context and explaining its relevance to the overall conclusions of the risk assessment does not serve the purpose of the assessment, which is to provide as accurate a picture as possible of the potential for adverse impact in order to allow for informed decision-making.

## Concluding comments

Plantation forests are highly managed ecosystems. While they provide important ecosystem services, the primary purpose of such forests is to produce wood and other forest products. Much is known about the biology of the tree species used in plantations, and the site-specific effects of silvicultural practices on productivity, reproduction and growth. The fact that plantation forests are intensively managed means that there is significant familiarity with and knowledge about both the host plant and the receiving environment, and this knowledge is fundamental to a robust ERA.

The ERA paradigm that has been successfully applied to the precommercial evaluation of GE crops is equally applicable to the risk assessment of GE trees that will be used in plantation forestry. While the biology of forest trees differs from annual row crops, characteristics of trees such as longevity, size and scale are manageable and do not preclude the evaluation of GE trees for deployment in confined field trials or forest plantations. Regulatory authorities in countries such as Australia, Canada, Japan and the US have approved the unconfined release of other long-lived perennial species such as transgenic alfalfa, rose, plum and papaya, and most governments have related experience in the risk assessment of nontransgenic, introduced perennial species that is highly relevant to the ERA of forest tree species.

ERA for confined field trials of GE trees must be distinguished from ERA for unconfined releases. The emphasis for risk assessment in relation to confined field trials is the expected effectiveness of confinement measures designed to minimize environmental exposure. Biological information relevant to effective confinement primarily consists of knowledge about the characteristics of the tree's reproductive biology, the effect that the transgenic trait is anticipated to have on those characteristics and the presence of sexually compatible species in proximity to the trial site.

Confined field trials of experimental GE trees have been approved in a limited number of countries (see Table 1) often with the condition that the trial is terminated before the trees reach sexual maturity. While this is a practical measure designed to limit exposure, it also eliminates the opportunity to evaluate GE trees expressing traits of relevance to later stages in the life cycle. There are examples where GE trees in field trials have been allowed to reach sexual maturity and flower, allowing meaningful data from older trees to be collected (e.g. APHIS, 2007b, 2008; CTNBio, 2007). It should not be assumed *a priori* that confinement necessarily requires the prevention of flowering or the ability of the tree to reach full maturity or full rotation. In order to successfully complete an ERA for the unconfined release of a GE forest tree, evaluation through sexual maturity will be important for a number of tree species × trait combinations. There are ways that this can be achieved by using appropriate confinement and mitigation protocols without compromising field test design or environmental safety. The completion of over 700 field trials in a range of forest tree species over the past two decades, with no adverse environmental impacts is testimony that processes already in place for ERA of confined field trials of trees are effective.

ERA of GE trees for unconfined release focuses on the behaviour and interactions of the GE tree in the anticipated area(s) of deployment. Information about host biology and the receiving environment is supplemented with additional data accrued from laboratory tests and studies undertaken during confined field trials. In some cases, such as the transformation of a tree species with a gene that has already undergone significant evaluation in one or more crop species, those data may come from the literature and/or other risk assessments, and does not need to be repeated.

## Supporting information

**Figure S1** Stages in GE plant development from research through to cultivation with concurrent regulatory requirements.
