# Supplementary figures and images for: Genetically engineered trees for plantation forests: key considerations for environmental risk assessment
Source: Plant Biotechnol J. 2013 Aug 5;11(7):785–98. doi: 10.1111/pbi.12100 (PMC3823068; doi:10.1111/pbi.12100)

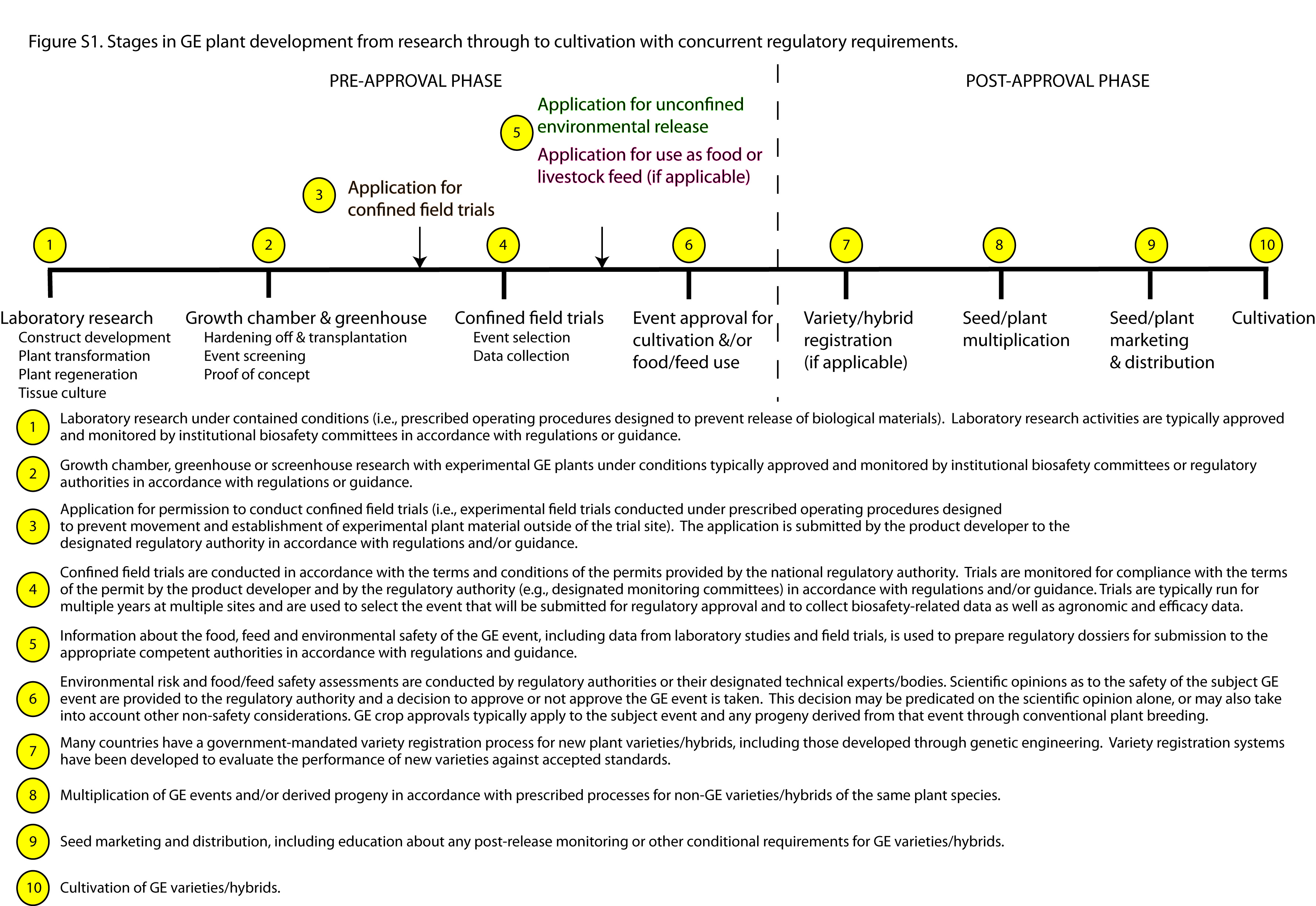

Supplement: Supplementary file 1 [file pbi0011-0785-SD1.jpg]
